# Metasurfaces-based imaging and applications: from miniaturized optical components to functional imaging platforms

**DOI:** 10.1039/c9na00751b

**Published:** 2020-01-15

**Authors:** Dasol Lee, Junho Gwak, Trevon Badloe, Stefano Palomba, Junsuk Rho

**Affiliations:** Department of Mechanical Engineering, Pohang University of Science and Technology (POSTECH) Pohang 37673 Republic of Korea jsrho@postech.ac.kr; Department of Chemical Engineering, Pohang University of Science and Technology (POSTECH) Pohang 37673 Republic of Korea; Institute of Photonics and Optical Science, School of Physics, The University of Sydney Sydney NSW 2006 Australia; The University of Sydney Nano Institute, The University of Sydney Sydney NSW 2006 Australia

## Abstract

This review focuses on the imaging applications of metasurfaces. These optical elements provide a unique platform to control light; not only do they have a reduced size and complexity compared to conventional imaging systems but they also enable novel imaging modalities, such as functional-imaging techniques. This review highlights the development of metalenses, from their basic principles, to the achievement of achromatic and tunable lenses, and metasurfaces implemented in functional optical imaging applications.

## Abstract 2

This review focuses on the imaging applications of metasurfaces.

## Graphical abstract

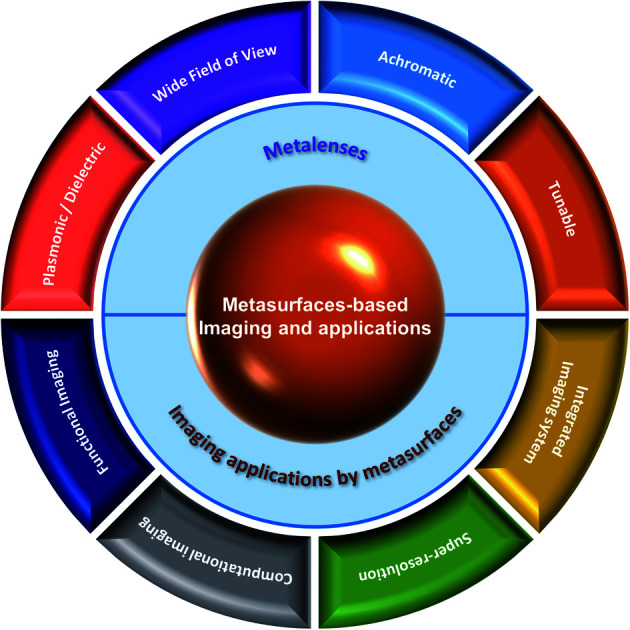

## 1. Introduction

Optical metamaterials are materials that are artificially engineered to exhibit optical properties that are not found in conventional materials. Optical metamaterials have exceptional advantages in realizing optical functionalities, and therefore have been used in many applications, including negative refraction,^1–3^ super-resolution imaging,^4–10^ optical cloaking^11,12^ and photonic topological metamaterials.^13–16^ Although metamaterials have shown superior capabilities in controlling light, multiple stacks of the unit cell are required, complicating nanofabrication and creating additional channels where optical losses take place.

In order to overcome such drawbacks, metasurfaces, *i.e.* two-dimensional metamaterials, have been actively investigated. Subwavelength scatterers, called meta-atoms, are engineered to modulate the properties of an incident optical wave, such as its phase distribution, its intensity and its polarization state, giving rise to a scattered optical wave with substantially different optical properties. Thus, various applications of wave modulation can be realized with metasurfaces, such as metaholograms,^17–23^ wavefront shaping,^24–26^ and structural color printing.^27–30^ Metalenses and the application of metasurfaces in imaging systems have been widely studied thanks to their 2D nature and potential for replacing and improving optical components.

In this review, we mainly focus on the progress of applications that use metasurfaces. First, we will describe what metalenses are, then discuss achromatic and tunable metalenses. Afterwards, we will review novel applications of metasurfaces in optical systems, such as super-resolution imaging, computational imaging and other functional imaging techniques. We will conclude this review by suggesting perspectives for future research on metasurface-based imaging techniques.

## 2. Metalenses

This section introduces the working principle of metalenses. Two kinds of metalens, plasmonic and dielectric, are examined, and various designs for achieving a wide field of view (FOV) and overcoming chromatic aberration are also discussed, along with how they can be optimized. Lastly, the emerging field of tunable metalenses and their current progress are briefly presented.

### 2.1 Phase profile control

Metalenses use metasurfaces, which are 2D arrays of subwavelength-spaced optical scatterers, and can work in either reflection or transmission. They do not rely on propagation effects such as refraction, reflection or diffraction of light, as conventional optics do, and therefore circumvent the bulkiness, weight and expense of conventional optics. Metasurfaces can give abrupt, spatially-varying phase changes to incident light, and can shape its wavefront in accordance with the spatial distribution of the optical scatterers.^31^ Adjustment of the geometrical parameters (*e.g.* shape, size, orientation) of the metasurface building blocks (MBBs) allows almost unrestricted freedom in engineering the optical properties of a reflected or transmitted wavefront, and therefore control of the optical wave behavior with subwavelength resolution. Metasurfaces are free from spurious high diffraction orders that commonly occur in conventional diffractive components such as gratings.^31^ These spurious diffraction orders degrade the efficiency of diffractive components and cause undesirable phenomena such as virtual focal spots, halos, and imaging artifacts. Once the required phase variations are digitized for each MBB on a metasurface to focus incident light on a single point, they can be fabricated using standard lithography techniques. Light is dictated by the parameters of the MBBs and the refractive indices of the surrounding media.^24^ The hyperbolic phase profile required to focus a collimated optical wave orthogonal on a metasurface can be expressed as follows^32^1
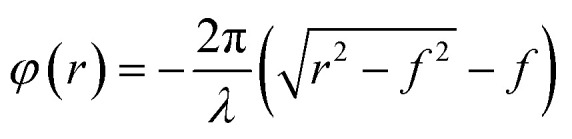
where *f* is the focal length and *r* is the radial position from the center and *λ* is the wavelength of the optical wave. As long as the incident light arrives normal to the flat metasurface, the reflected or transmitted wavefronts remain spherical. Hence, metasurface-based flat lenses are free from spherical aberration.^32^

In order to better illustrate the relationship between the phase change and the geometrical parameters of the building block (BB), consider a square dielectric pillar with width *W* and height *H*, located on a square lattice with lattice constant *U*.^31^ The lattice acts as a single element and its effective refractive index *n*_eff_ can be defined by modulating *W*, since it controls the confinement of light. The extent to which the pillar fills the lattice is referred to as the fill factor (FF). If FF = 0, the *n*_eff_ is equal to that of the surrounding medium; if FF = 1, the pillar fills the entire lattice, and *n*_eff_ is equal to the bulk refractive index of the material. The accumulated phase shift due to the propagation of light through a single lattice can be expressed as2
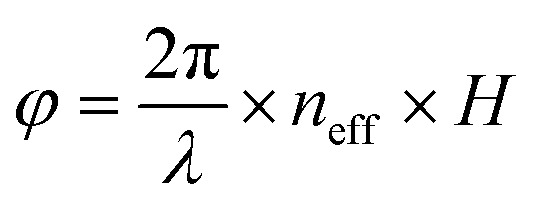
where *λ* is the wavelength of the incident light. To achieve the full phase difference of 2π between unit lattices with FF = 0 and 1, the height must be3
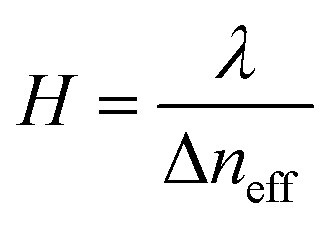
where Δ*n*_eff_ = *n* − 1. If a dielectric with *n* > 2 (Δ*n*_eff_ > 1) is to be used for the BB material, *H* becomes comparable to the incident wavelength,^31^ which provides an intuitive design criterion for the MBB.

### 2.2 Plasmonic metalenses

Metalenses exploit the manipulation of the optical properties of light by the MBBs. One of the most commonly used techniques is to make use of plasmonic antennas.^33^ As light impinges on the antenna, localized surface plasmons are set up around the surface. The antenna exhibits optical resonance when the antenna's lateral size is approximately half the surface plasmonic wavelength. Under this condition, the incident light is in phase with the coherent oscillation of the free electrons and therefore a current is generated from which light scattering originates. Therefore, varying the antenna's geometrical parameters such as size, shape and orientation can induce abrupt phase changes of the scattered light over distances much smaller than *λ*.

Plasmonic metalenses are also promising candidates for flat lenses. Surface Plasmon Polaritons (SPPs) excited by nano-sized holes can be focused into a subwavelength-sized spot with high near-field intensity.^34^ Theoretical and experimental reports have shown that near-field focusing of surface plasmons can be achieved with different antenna shapes such as circular and elliptical structures.^35,36^ Far-field focusing in the visible light spectrum with quasiperiodic nanohole arrays embedded on metal screen has also been reported.^35^

**Fig. 1:**
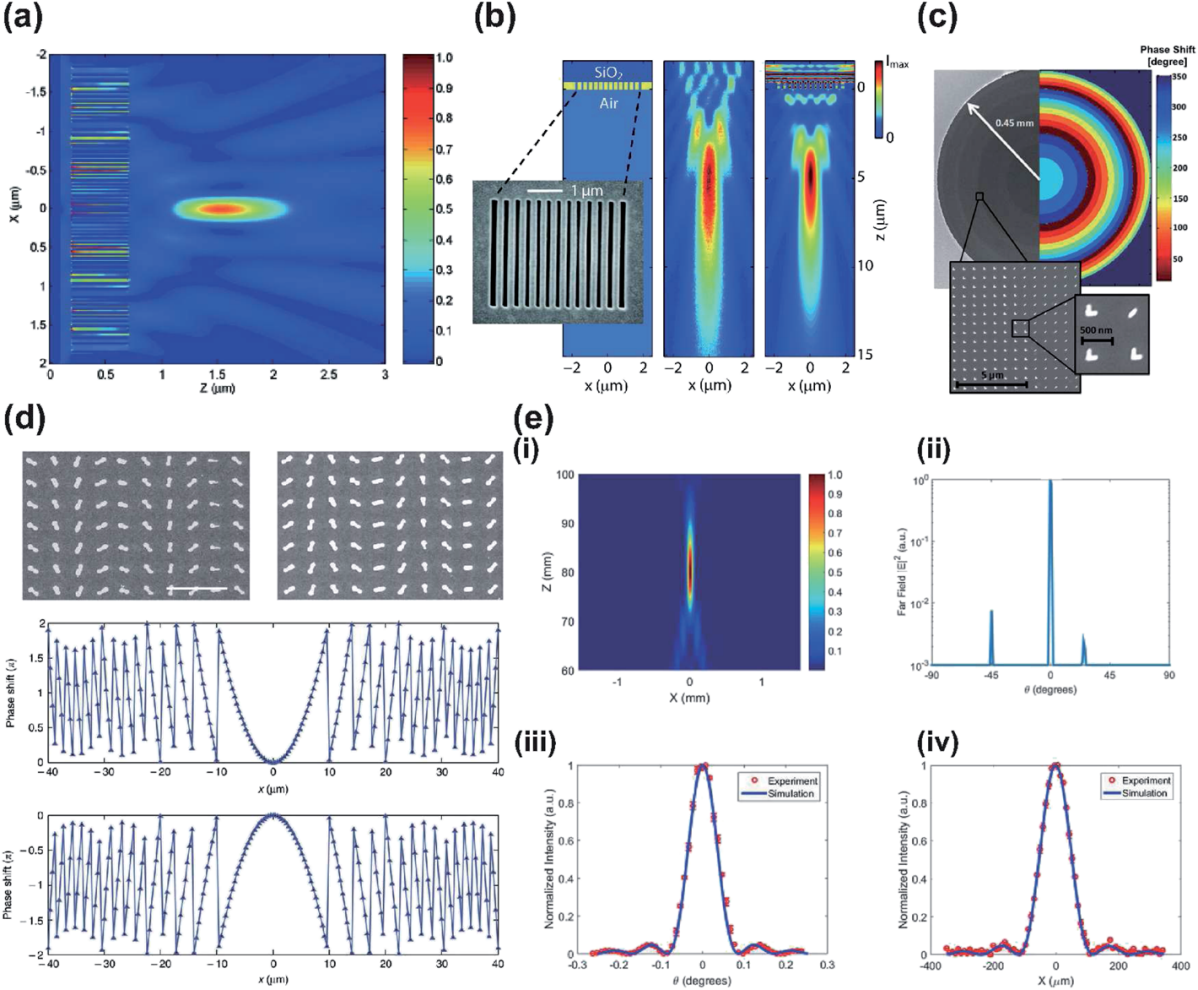
(a) FDTD simulation of normalized Poynting vector S_*z*_ for designed metallic nano-slit lens. Film thickness is 500 nm, and the total number of slits is 65. (b) Geometry of the metalens (left) with 400 nm optically thick gold film with air slits of varying widths milled on a fused silica substrate (dark blue). Inset: SEM image of air slits. Measured (center) and simulated (right) focusing pattern at the design wavelength of 637 nm. (c) SEM image of the fabricated lens with 3 cm focal length (left). The corresponding phase shift profile (right) is radially discretized according to the phase shifts of the eight antennas. Insets: close-ups of patterned antennas. (d) SEM image of plasmonic metalens on glass substrate with negative polarity (top left) and positive polarity (top right) for incident light with RCP. Expected phase shift by positive (middle) and negative (bottom) lens, for RCP incident light. (e) (i) Focal spot intensity distribution of the reflected beam in the *x*–*z* plane, with antenna arrays centered at *x* = 0. (ii) Most of the reflected light beam in far-field projection was focused at 0°, normal to the metalens surface. Simulation and experimental results of (iii) angular scan and (iv) translational scan of the normalized reflected beam intensity at the focus. (a) Reprinted with permission from ref. 38. Copyright 2005 The Optical Society. (b) Reprinted with permission from ref. 39. Copyright 2008 American Chemical Society. (c) Reprinted with permission from ref. 41. Copyright 2012 American Chemical Society. (d) Reprinted with permission from ref. 44. Copyright 2012 Nature Publishing Group. (e) Reprinted with permission from ref. 49. Copyright 2016 The Optical Society.

The previous work exploited amplitude modulation and appropriate spatial distribution of metallic apertures. For metalenses based on amplitude modulation, all scatterers must ideally have the same maximum forward-scattering amplitudes and negligible optical absorption loss in order to exhibit diffraction-limited focusing with high efficiency; this can impair the implementation of plasmonic metasurfaces since they have intrinsically high optical absorption. A theoretical alternative, to mitigate this issue, is generating optical focusing by phase modulation, which can be achieved by controlling the depth of antenna and width of nano-slit array.^37^ Numerical simulations of nano-slit arrays with constant depth and varying width (Fig. 1a) have theoretically shown the potential of implementing a phase-modulation method.^38^ Decreasing the width of the slit increases the local phase shift over the same propagation length. Phase modulation was also experimentally demonstrated (Fig. 1b) by using 400 nm-thick gold film with air slits of different widths (80 to 110 nm); the device showed ∼0.2π phase coverage.^39^

For an antenna that has a single resonance, an array of a V-shaped metallic antenna can cover a phase shift of 2π and a large range of scattering amplitudes; these capabilities are crucial for a full control of the wavefront.^24,40^ The V-shaped antenna have also been used to make spherical aberration-free flat lenses (Fig. 1c) in the near-infrared (NIR) range.^41^

Alternatively, Pancharatnam–Berry (PB) phase^42,43^ can be used to achieve 2π phase shift. The PB phase can be used to control the phase shift by rotating nanostructures that have identical sizes. Lenses that exploit the PB phase with convex and concave surfaces have been designed (Fig. 1d) with a 40 nm-thick gold antenna and an 80 × 80 μm^2^ aperture.^44^ Two lenses were used; for an incident wave with right circular polarization (RCP), one had a focal length of −60 μm, and the other had a focal length of +60 μm. The phase shift was reversed by using left circular polarization (LCP), which is an intrinsic property of a metalens that uses PB phase.^45^

Metalenses that exploit PB phase have also used a reflective scheme to achieve 2π phase shift, by rotating arrays of antenna separated from a metallic mirror by a dielectric spacer.^46,47^ This allowed the light polarization to be preserved and was exploited in the NIR^48^ and mid-infrared (MIR)^49^ regions. The latter work also showed high efficiency at diffraction-limited focusing (Fig. 1e) with single-step photolithography, which could ease the fabrication of large-diameter lenses.

Despite major progress in plasmonic metalenses, their efficiency in the visible and NIR wavelength ranges is capped at 25% by fundamental limitations;^50,51^ this limit combined with the high intrinsic optical loss^41^ impede their practical applications. To circumvent these limitations, metalenses based on dielectric metasurfaces have been investigated.^52,53^

### 2.3 Dielectric metalenses

Metalenses that use dielectric phase shifters can confine light in a subwavelength region with negligible loss; this trait has enabled new design and applications in meta-optics. When dielectric phase shifters are used as truncated waveguides with low quality factor, Fabry–Perot effects occur due to mismatch between the refractive indices of the dielectric waveguide (DW) ends and the surrounding medium. Also, when incident light impinges on the DW, some of the light is not coupled into waveguide modes and either propagates through the DW or radiates into the surrounding medium.^31^ Therefore, rigorous finite-difference time-domain simulations should be performed to assist the process of designing metalenses.

The spatial digitization of phase profile provided by adjacent MBBs must satisfy the following sampling criterion^31^4
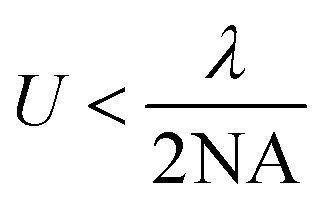
where *U* is the distance between two adjacent lattices, and NA is the numerical aperture. The criterion demonstrates a trade-off: as *U* decreases, the sampling quality increases and the phase change required between two MBBs decreases; the consequence is a decrease in the abruptness of the change in geometrical parameters of MBBs. Satisfying this criterion becomes increasingly difficult when the incident light's *λ* decreases or the required NA increases. This difficulty occurs because as *U* decreases, the DW width decreases and the required aspect ratio to achieve full phase coverage increases, so fabrication requirements become increasingly stringent. Conventional fabrication techniques have difficulty in meeting these requirements. Dry-etching^54^ lacks control over the geometry of DWs, such as angled sidewalls. Liftoff^55^ cannot provide enough height for full-phase coverage. Also, as the lateral size of DWs decreases, the optical confinement decreases and near-field coupling between DWs increases; this trend degrades focusing efficiency.

Plasmonic MBBs can easily meet the criterion because they can be closely packed, due to their deep-subwavelength confinement of the plasmonic modes. Dielectrics that have high refractive index can also fulfill this condition by increasing the light confinement and reducing the near-field coupling, so that the MBBs can be more closely packed.

**Fig. 2:**
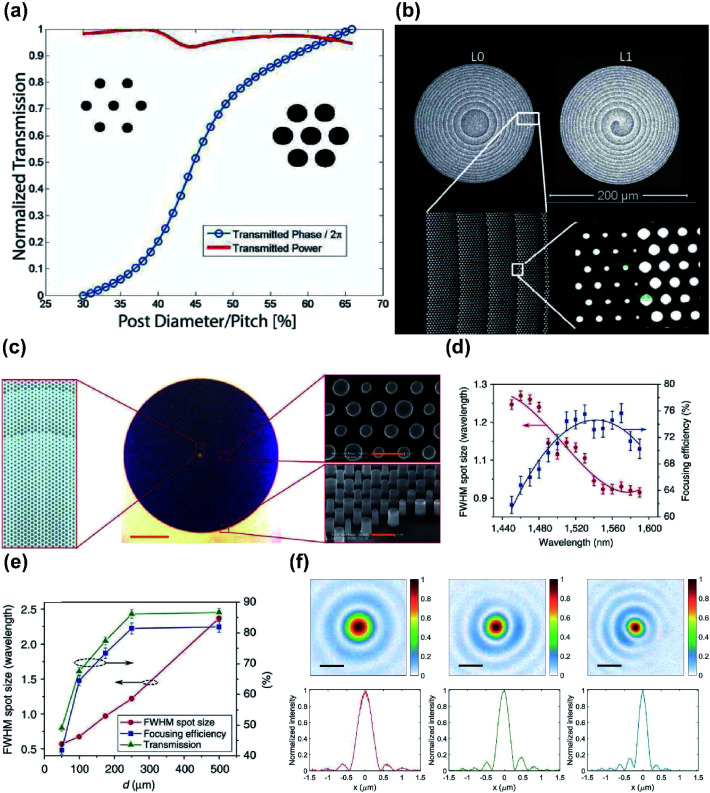
(a) High-contrast gratings (HCG) lens design and simulation at 850 nm. Simulation result of normalized transmission with a post height of 475 nm and various post diameters. (b) Fabricated HCG lenses with *l* = 0 focusing (top left) and vortex functionality (left). Bottom: SEM image of patterned meta-atoms. (c) Schematic (left) and optical microscope image of fabricated HCTA lens (middle). Right: SEM image of silicon posts on HCTA metalens. (d) Full width at half maximum (FWHM) spot size and focusing efficiency showing wavelength dependency. (e) Measured FWHM spot size, focusing efficiency, and transmission at varying focal length. (f) Measured focal spots at high NA = 0.85 at visible wavelengths of 660 (left), 532 (middle), and 405 nm (right), with corresponding focal-spot-normalized intensities. (a and b) Reprinted with permission from ref. 56. Copyright 2014 Institute of Electrical and Electronics Engineers. (c–e) Reprinted with permission from ref. 57. Copyright 2016 Nature Publishing Group. (f) Reprinted with permission from ref. 58. Copyright 2016 American Chemical Society.

For example, polarization-independent lenses with dielectric BBs of circular silicon (Si) posts in hexagonal arrays have been designed.^56^ Due to high transparency of porous Si in the NIR spectral region, the lenses focused the incident light of 850 nm into a spot of ∼10*λ* at a high transmission efficiency of 70% (Fig. 2a and b). However, the hexagonal configuration of BBs caused a high *U*, which limited the maximum achievable phase gradient of the lenses and thus the maximum NA.

A single silicon post can serve as an efficient MBB and provide full phase coverage.^57^ This MBB design uses high-contrast transmission arrays to maintain high transmission with subwavelength spatial resolution, and thereby enables large NAs. These lenses show focusing efficiencies up to 82% and focal spots as small as 0.57*λ* (Fig. 2c–e). Because of the weak coupling between MBBs, the lenses have small deviation from the designed phase due to near-field coupling and are free from spherical aberration.

High-precision lenses that use dielectric MBBs in the visible spectrum have been also demonstrated.^58^ These metalenses were fabricated using circular titanium dioxide (TiO_2_) posts that were fabricated using atomic layer deposition; they have a relatively high NA = 0.85 and efficiency > 60% at *λ* = 532 and *λ* = 660 nm (Fig. 2f), but at *λ* = 405 nm, the efficiency decreased to 33%, because the required small radius introduced fabrication difficulties. When NA was reduced to 0.6, the maximum focusing efficiency of 90% was achieved at the designed *λ* = 660 nm.

### 2.4 Wide field of view

Although diffraction-limited focusing with high NA is a key ingredient in the evaluation of imaging resolution, various other types of aberration, such as chromatic, coma and astigmatism, must be considered in practice. Off-axis aberration or wide field of view (FOV) is especially one of the major and essential features of modern imaging systems. In conventional optics, relatively high FOV is achieved by cascading several layers of bulk lenses that are manufactured separately using cutting, polishing, and grinding processes. However, these processes introduce stringent alignment tolerances and makes the entire system heavy, bulky and costly, so this approach is not suitable for optical miniaturization. In contrast, the unique properties of metasurfaces, including lithographic alignment of optical systems, capacity of engineering a phase gradient, which can be tailored based on the required functionalities, and micron-level thickness, can be exploited to achieve a wide FOV. Off-axis aberration by a metalens may be corrected if it is manufactured on the surface of a sphere.^59–61^ However, direct patterning of MBBs on a curved surface is challenging. A conformal metasurface^62^ has been suggested as another solution but the resulting surface may not be flat.

**Fig. 3:**
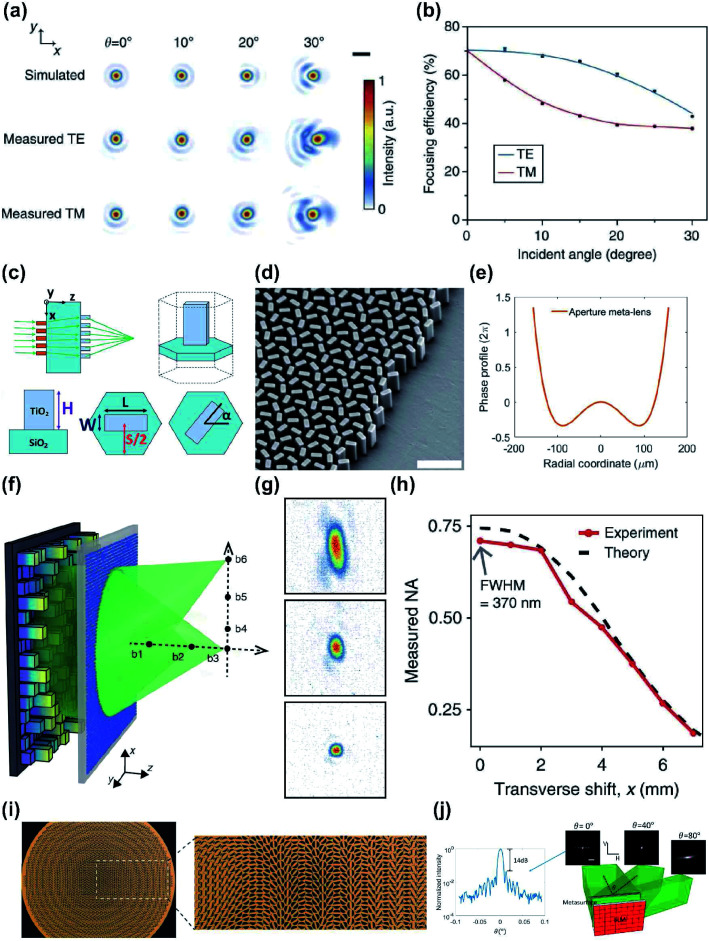
(a) Simulated and measured focal-spot intensity profiles at different incident angles *θ*. (b) Focusing efficiency of both transverse electric (TE) and transverse magnetic (TM) decrease as incident angle increases. (c) Schematic of metalens doublet design with its meta-atom configuration. (d) Side-view SEM image of the focusing metalens. (e) Phase profile of the aperture metalens to correct positive and negative spherical aberrations. (f) Schematic of diffraction-limited focusing over an extended volume. (g) Transversely shifted foci of the metasurface in (f) at *x* = 7 mm (top), 4 mm (middle), 1 mm (bottom), scanned on the fixed focal plane at *z* = 3.8 mm. (h) Measured and theoretical NA of the foci created with change in transverse shift along the *x* axis. (i) Transversal catenary field formed within the unit cell at 19 GHz. (j) Normalized intensities against steering angles and the far-field beam profile at the steering angles of 0°, 40°, and 80°. (a and b) Reprinted with permission from ref. 63. Copyright 2016 Nature Publishing Group. (c–e) Reprinted with permission from ref. 64. Copyright 2017 American Chemical Society. (f–h) Reprinted with permission from ref. 65. Copyright 2018 Nature Publishing Group. (i) Reprinted with permission from ref. 67. Copyright 2018 John Wiley & Sons, Inc. (j) Reprinted with permission from ref. 69. Copyright 2018 The Optical Society.

The use of a correcting layer may be a way to obtain a wide FOV.^63,64^ Doublet metalenses are constituted by a correcting and a focusing lenses cladded by a layer of cured SU-8 polymer.^63^ Two lenses are applied on opposite sides of a glass substrate, according to their functionalities. These lenses provided nearly diffraction-limited focusing at the design wavelength *λ* = 850 nm (NIR) over a FOV of 30° (Fig. 3a and b). A similar design with a FOV of 25° has been achieved in the visible range at the design wavelength of *λ* = 532 nm.^64^ These lenses achieved diffraction-limited focusing with an NA of 0.44 and a focal length of 342.5 μm (Fig. 3c–e).

Disorder-engineering metasurfaces were used to demonstrate a single-layer metalens with improved resolution and wide FOV.^65^ In the context of addressable focal spots assisted by disorder-engineering metasurface, the input and output field are controlled by the spatial light modulator (SLM). The input and output field each have spatial dimensions *N* and *M* which are primarily determined by the number of SLM pixels. Therefore, the disorder-engineering metasurface which converts the input field into output field is defined to have a transmission matrix *T* with *P* elements, where *P* = *M* × *N*. Typically, disordered media have optical randomness, so they need extensive characterization of input/output responses, a rigorous process of defining *P* elements.^65^ For practical applications, *P* > 10^12^ is desirable. The highest *P* value reported to date is ∼10^8^, during a measurement of 40 s.^66^ However, rather than engineering the statistical properties of the scattered light pattern in a conventional disordered medium, individual input–output responses can be tailored in disordered metalenses, meaning that the exhaustive characterization process can be reduced to simple alignment problems. When disordered metalenses are combined with the SLM and compared with the same SLM used alone (NA = 0.033) (Fig. 3f–h), the disordered metalenses showed diffraction-limited focusing with NA of 0.95, 0.9, and 0.75 along the optical axis at a distance *z* = 1.4, 2.1 and 3.8 mm, respectively. As the focal point is moved laterally along the focal plane at *z* = 3.8 mm, the spot size along the *x* axis increases from 370 to 1500 mm. The total number of resolvable points is estimated to be ∼4.3 × 10^8^ in a FOV of ∼8 mm.

Perfect conversion from rotational symmetry to translational symmetry in the light field has been presented as another approach to attain a larger FOV.^67^ To achieve this conversion, the following should be satisfied:

5 *k*_0_ sin *θ*_*x*_*x* + *k*_0_ sin *θ*_*y*_*y* + *φ*_m_(*x*,*y*) = *φ*_m_(*x* + *Δ*_*x*_ + *Δ*_*y*_)

where *k*_0_ is the wavenumber in free space, *φ*_m_(*x*,*y*) is the phase shift profile that the flat lens exhibits, *Δ*_*x*_ and *Δ*_*y*_ are the translational shifts of *φ*_m_(*x*,*y*) at incidence angles of *θ*_*x*_ and *θ*_*y*_ respectively. In order to validate this method, the authors designed catenary-inspired PB-phase unit cells and arranged them in a hexagonal lattice where each rotating metallic cut-wire is located at the center of a circular aperture (Fig. 3i).^68^ A simulation of a metalens with a diameter of 350 mm and focal length *f* = 87.5 mm (NA ∼ 0.89) was conducted. This showed a 60° FOV with relatively high efficiency (>80% when the incidence angle is tilted by 60°). This was achieved by transversely changing the location of the nanoantennas within the range of [−75.8 mm, 75.8 mm].

A new phase-array method has been reported;^69^ it also uses a disorder-engineered metasurface consisting of a waveguide made up of subwavelength-sized scatterers (square silicon nitride (Si_*x*_N_*y*_) nanoposts with height = 630 nm) deposited on fused silica. When aligned with an SLM, the disordered metalens could scatter light uniformly within a range of ±90° (Fig. 3j).

### 2.5 Control of chromatic dispersion

A metalens requires a phase-profile gradient, which is primarily determined by eqn (1) in order to focus incident light with certain focal length. This relationship implies that a 0 to 2π phase coverage can only be controlled by changing the geometrical parameters of each MBB or by rotating them. However, a metalens inevitably has different focal lengths at different designed wavelengths, hence chromatic aberration occurs.^70^ Such chromatic dispersion significantly limits a metalenses' usefulness in applications such as imaging and displays, which require broadband wavelength capabilities. In order to address this issue, metalenses have been designed to have invariant focal lengths at discrete wavelengths.^71–73^ The design principle of these multi-wavelength metalenses relies on integrating metalenses that work at different wavelengths into the same platform. This is achieved by using multilayers,^74^ multiplexing,^75–77^ or by exploiting spin–orbit interaction.^78^ However, these approaches are valid only for applications that need metalenses that operate at several discrete wavelengths, such as optical communication and fluorescence microscopy. To achieve continuous broadband effectiveness, the design complexity dramatically increases. Furthermore, unintended coupling and interference between metalenses occur, which degrades the overall efficiency.^70^ Therefore, to achieve metalenses that work in over continuous broadband wavelengths, chromatic aberration must be completely eliminated.

**Fig. 4:**
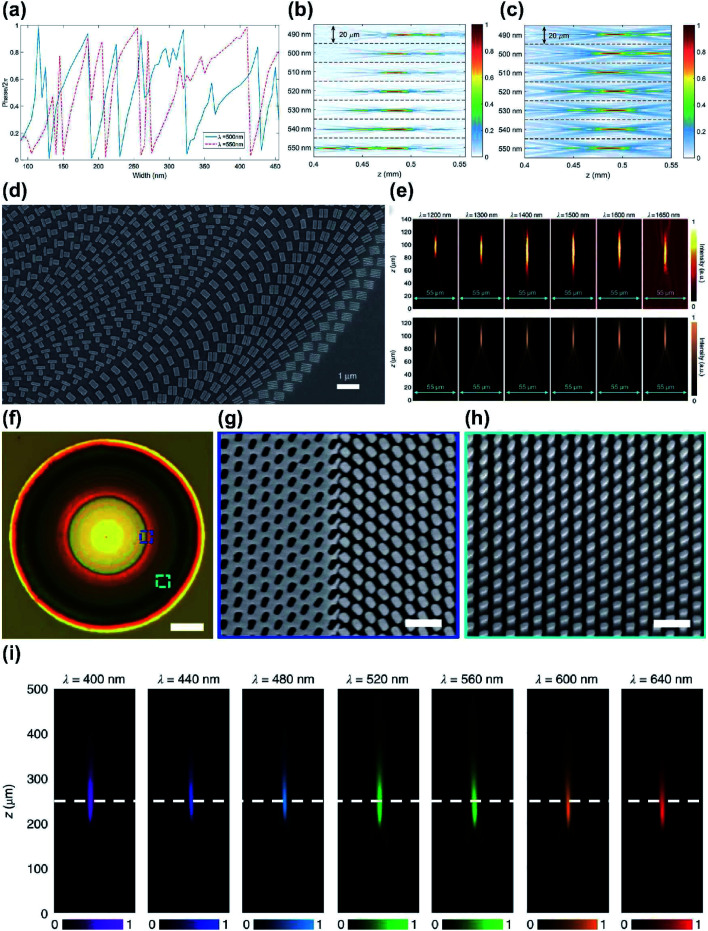
(a) Simulated phase shift in reflective mode as a function of nanopillar width at wavelengths of 500 and 550 nm. (b) Measured and (c) simulated intensity profiles of the reflected beam at different wavelengths. In the wavelength range from 490 to 550 nm, variation of focal lengths is negligible with a standard deviation of 2.7 μm. (d) SEM image of fabricated metalens. (e) Experimental (top) and numerical (bottom) horizontal cut intensity profiles of BAML at various incident wavelengths. (f) Optical image of the fabricated BAML with NA = 0.106. SEM images of (g) boundaries between nanopillars and identical apertures in the inner square in (f), and (h) nanopillars in the outer square in (f). (i) Measured light intensity profiles of BAML at various visible incident wavelengths. (a–c) Reprinted with permission from ref. 79. Copyright 2017 American Chemical Society. (d and e) Reprinted with permission from ref. 80. Copyright 2017 Nature Publishing Group. (f–i) Reprinted with permission from ref. 82. Copyright 2018 Nature Publishing Group.

Several metalens designs have used reflective mode, because it extends the propagation length, and thereby increases phase coverage, and achieves large reflection amplitudes.^70^ An achromatic metalens (AML) composed of TiO_2_ nanopillars separated from a metallic mirror by a dielectric spacer has been demonstrated experimentally.^79^ Manipulation of guided mode resonances gives each phase shifter both multiple 2π phase coverage, and anomalous dispersion (Fig. 4a). A reference phase was used to minimize the difference between the required and implemented phase simultaneously for all selected wavelengths. The resulting AML had a uniform focal length of 485 μm at 490 ≤ *λ* ≤ 550 nm, giving an NA = 0.2 (Fig. 4b and c).

Reflective broadband achromatic metalenses (BAML) working in NIR bandwidth from 1200 to 1680 nm were proposed by using integrated-resonant units (IRUs) in PB phase.^80^ The authors first set maximum wavelength *λ*_max_ and minimum wavelength *λ*_min_, then reconstructed the original spatial phase-gradient equation^80^6
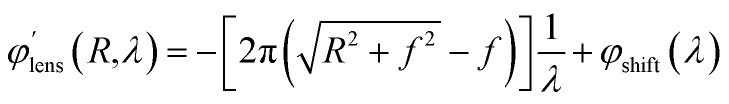
where *φ*_shift_(*λ*) corresponds to the phase dispersion with the inverse of wavelength. Therefore, *φ*_shift_(*λ*) must match the form 
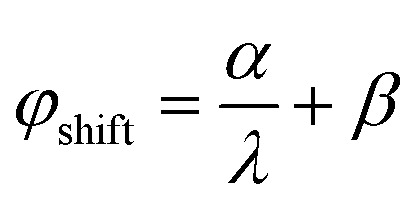
, where 
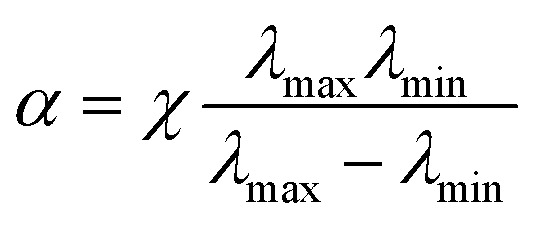
, 
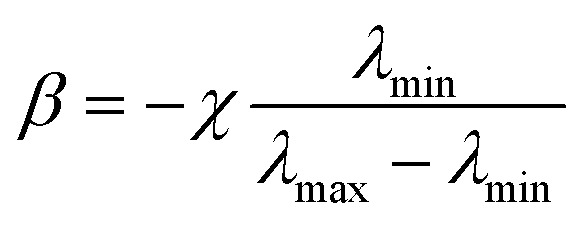
 and *χ* is the largest additional phase shift between *λ*_max_ and *λ*_min_ at the center of the metalens. *χ* is a key parameter in determining the diameter of the BAML. The BB consists of specially-arranged coupled gold nano-rods on a gold (Au) back reflector with silicon dioxide (SiO_2_) in between as the spacer (Fig. 4d and e). While metallic nano-rods support multiple plasmonic resonances such as fundamental dipolar mode and various high order modes, they manipulated the gap size and the number of resonators to generate smooth and linear phase changing slope between each resonances, termed as integrated-resonant state.^80^ Their IRUs building blocks provide design flexibility as larger phase compensation due to more radical phase dispersion can be directly realized by adding more resonators in the unit cell. A similar design that used aluminium (Al) nanorods was applied to obtain an AML that functions in visible wavelengths.^81^ Al has lower absorption loss than Au, so the designed BAML exhibits 20% efficiency over the working bandwidth and uniform focal length over 400 ≤ *λ* ≤ 667 nm.

While reflective mode AMLs effectively show broadband achromatic functionality, transmissive optical components are preferred in terms of practical applications. Metallic meta-atoms can be made very thin (*i.e.* with a low aspect ratio) and can support strong near-field coupling, but the intrinsic loss and the need for a back reflector make them unsuitable for a transmissive-mode AML. A transmissive-mode BAML has been demonstrated,^82^ using IRUs based on gallium nitride (GaN). Coupled dielectric resonators lack near-field coupling between resonators but can be functionalized as a truncated waveguide that has multiple Fabry–Perot resonances, with high efficiency and near-zero optical loss. Therefore, the authors used GaN nanopillars with high aspect ratios, and apertures as complementary structures to induce large phase compensation. The resulting BAML showed uniform focal length at 400 ≤ *λ* ≤ 660 nm with an average efficiency of 40% (Fig. 4f–i).

### 2.6 Tunable metalenses

Metalenses can be tuned to achieve active control over functionalities such as focal length. Tunable metalenses are desirable in applications where optical versatility is required. Up to now, only two approaches in designing tunable metalenses have been reported.

**Fig. 5:**
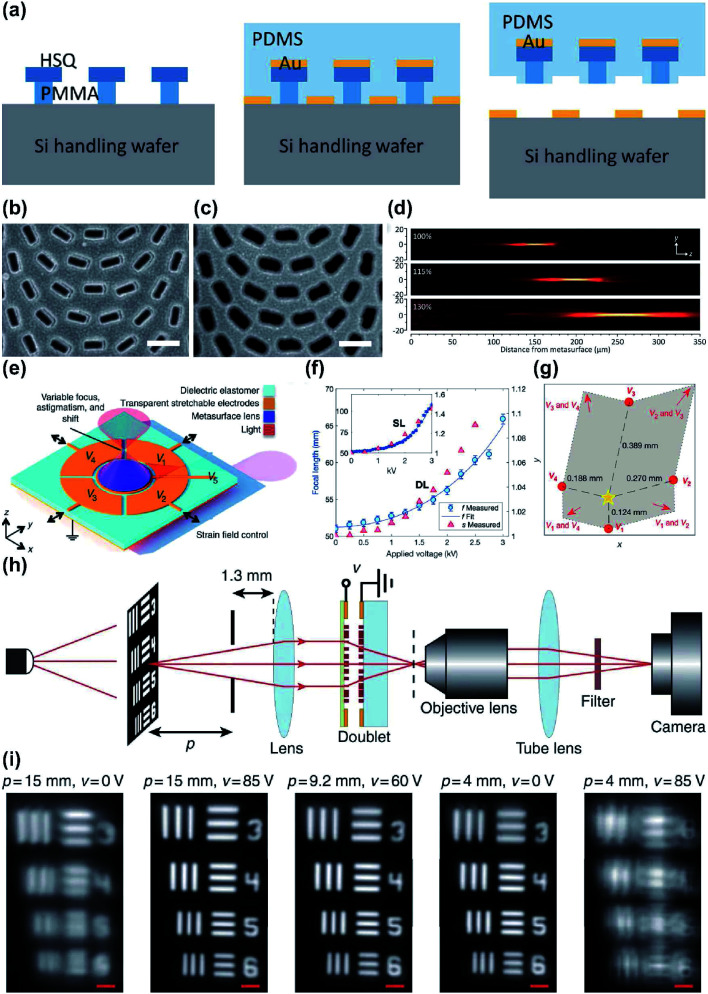
(a) Schematic of the fabrication process of tunable metasurface on PDMS substrate. SEM images of the Si handling wafer (b) before and (c) after PDMS curing and stripping. (d) Measured longitudinal beam intensity profiles generated by the tunable metalens stretched by 0% (top), 15% (middle), and 30% (bottom). (e) Schematic of tunable metalens with bias provided by five addressable electrodes to control the strain field of the metasurface. (f) Measurement of focal length and strain along with voltage applied at the center electrode V_5_ for double-layer (DL) and single-layer (SL) devices. (g) Three-dimensional shifting of focal spot in accordance with voltage applied to different electrodes. Yellow star: original focal spot. (h) Schematic of experimental apparatus using a regular glass lens and tunable metalens doublet. (i) Tuning of test image by applying adequate voltage for different imaging distances. (a–d) Reprinted with permission from ref. 85. Copyright 2016 American Chemical Society. (e–g) Reprinted with permission from ref. 86. Copyright 2018 The American Association for the Advancement of Science. (h and i) Reprinted with permission from ref. 87. Copyright 2018 Nature Publishing Group.

The most common approach is to use reconfigurable metalenses,^83,84^ where the physical dimensions such as the interval between meta-atoms and the geometrical parameters of individual meta-atoms can be varied. A change in the physical dimensions affects the near-field interaction between meta-atoms and hence the spatial phase gradient. This allows the reconfiguration of the entire metalens output wavefront. For example, metasurfaces on a stretchable substrate such as polydimethylsiloxane (PDMS) can be used as a platform for reconfigurable metalenses. By gradually changing the strain *ε* applied to the stretchable substrate, the phase profile changes proportionally by 1/(1 + *ε*)^2^. Stretchable metalenses are fabricated in two steps: nanofabrication of a metalens on a template, followed by the transferring of the pattern. For example, ∼30% linear change of the substrate in a mechanically-reconfigurable metasurface modifies the focal length from 150 to 250 mm in visible spectral region (*λ* = 632.8 nm).^85^ The metalens was fabricated first by using e-beam lithography and subsequent oxygen plasma etching to define hydrogen silsesquioxane and a polymethyl methacrylate (HSQ/PMMA) bilayer mask on a silicon substrate. Secondly, the poor adhesion between PMMA and silicon was exploited to cast a PDMS layer. Subsequently, the stretchable metasurface was removed from the silicon substrate (Fig. 5a–d).

Electrically-stretchable metalenses can correct astigmatism in addition to changing the focal length in three dimensions.^86^ The metalenses consist of silicon nanoposts on tape (3M, VHB 4905) as the stretchable substrate. Before the metalens transfer, the substrate is patterned with a carbon nanotube layer as an optically transparent electrode. The substrate is a type of electroactive polymer; it contracts along the axis on which an external bias is applied. Therefore, the lattice dimensions on the metalenses can be manipulated by applying a bias to specific electrodes; this process allows local tuning of the phase profile (Fig. 5e–g).

A metalens doublet fabricated as a microelectromechanical system (MEMS) has been reported;^87^ its focal length can be tuned continuously. The BBs are amorphous-Si nanoposts, and the doublet is composed of a converging metalens on a movable SiN_*x*_ substrate and a diverging metalens on a fixed glass substrate. A spacer made of optical resist is inserted between them and both metalenses are surrounded by metallic rings. Bias can be applied through the metallic rings to actively control the interspace between the metalenses; this process allows dynamic variation of the effective focal length (Fig. 5h and i).

The second approach is to use an active material with controllable optical properties in metalenses. Active materials such as gallium arsenide^88^ and phase-change materials^89–92^ can be tuned at will by applying external excitation. Phase-change materials are strong candidates to be utilized in tunable metalenses, because of their low optical loss from the visible to the MIR spectral regions, and because they can change phase quickly.^93^ Phase-change materials have large optical contrasts between amorphous and crystalline states, which can be reversibly switched by collimating laser-electrical pulses on them. For example, a tunable metalens doublet based on PB phase has been designed to operate in the MIR regime.^94^ The metalens consists of two layers of Au nanorods of different lengths on a phase-change thin film. When the phase-change film is in the amorphous state, the metalens with long nanorods is in resonance and the metalens with short nanorods is off resonance; the resonances are exchanged when the film is switched to the crystalline state. The two metalenses have different phase profiles and thus different focal lengths. Therefore, switching the state of the phase-change film changes the focal length of the metalens doublet. Multilayer metalens that use imbedded phase-change material can have as many focal lengths as the number of metalenses.

## 3. Imaging techniques and applications by metasurfaces

This section introduces optical systems or imaging techniques that use metasurfaces. Metasurfaces are compact and have advantages in controlling light, and therefore have the potential to replace existing optical systems. Metasurfaces have been widely applied in various functional imaging applications such as super-resolution techniques, computational imaging, polarization imaging and hyperspectral imaging.

### 3.1 Metalenses integrated in imaging systems

Metalenses have been successfully integrated into state-of-the-art optical systems such as confocal microscopes, two-photon microscopes and optical coherence tomography (OCT) systems. In biological science, these optical imaging methods are widely used and commercialized as fluorescence imaging or non-invasive bio-imaging tools for diagnosis and observation.

**Fig. 6:**
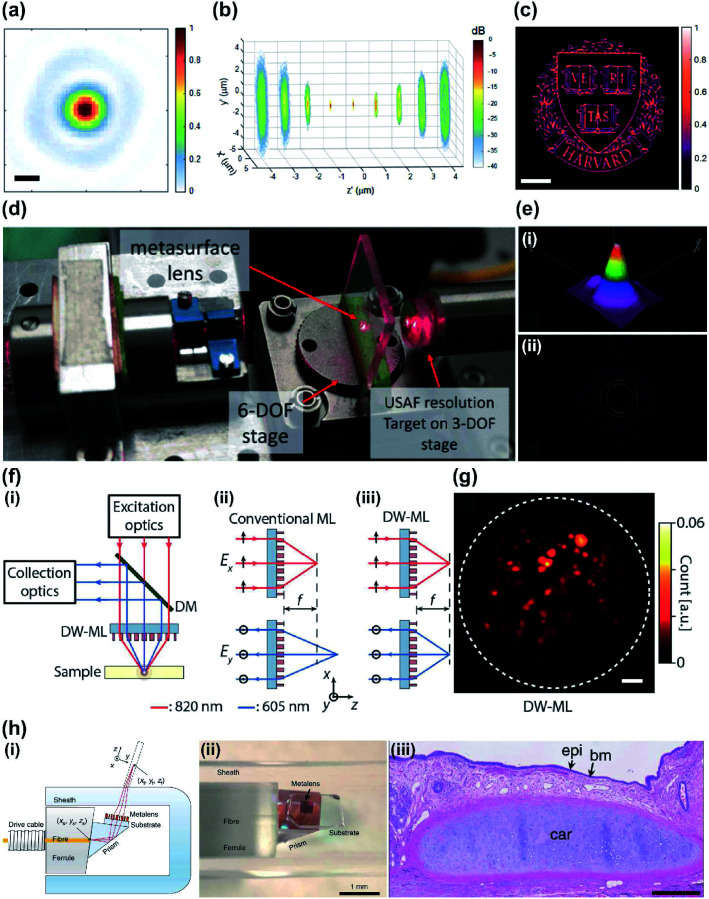
(a) Normalized intensity profile of focused beam of metalenses used in confocal microscope with NA = 1.1 (scale bar: 200 nm). (b) dB-scale intensity distribution of focused beam along *z*-axis (from −4 μm to 4 μm). The designed metalens works at 532 nm. (c) Confocal imaging result with oil immersion metalens. The size of captured image is 60 μm × 60 μm (scale bar: 10 μm) (d) schematic of fiber-based laser confocal microscope with metalens. (e) (i) 3D intensity profile and (ii) 2D intensity profile of focused beam from metalens with monochromatic metalens at 660 nm. (f) (i) Schematic of two-photon microscope that uses a metalens. The metalens focuses laser beam into a sample and collects the emitted fluorescence signal. (ii) Schematic of conventional metalens and (iii) double-wavelength metalens wavelengths 605 nm and 820 nm. Double-wavelength metalens shows same focal length *f* regardless of wavelengths. (g) Acquired two-photon microscope image of fluorescence beads (scale bar: 10 μm). (h) (i) Schematic of metalens-implemented OCT system and (ii) photograph of the tip of the endoscope. (iii) Captured endoscopic images of human lung resections. (a–c) Reprinted with permission from ref. 95. Copyright 2017 American Chemical Society. (d and e) Reprinted with permission from ref. 96. Copyright 2018 Institute of Electrical and Electronics Engineers. (f and g) Reprinted with permission from ref. 100. Copyright 2018 American Chemical Society. (h) Reprinted with permission from ref. 101. Copyright 2018 Nature Publishing Group.

A confocal microscope that uses a metalens exploits the small size and light-focusing ability of metalenses demonstrating superior imaging performance (Fig. 6a–c).^95^ A fiber-optic based laser scanning confocal microscope has shown performance that is comparable with that of conventional bulk optical laser scanning microscopes, while being compact and offering physical flexibility. An endoscopic confocal system has also been further miniaturized by using a monochromatic metalens (Fig. 6d) at *λ* = 660 nm.^96^ The designed Huygens metasurface has TiO_2_ resonators mounted in the confocal system. In practice, the metalens exhibits a full width at half-maximum (FWHM) spot size of 4.29 μm and a focal length of 4 mm (Fig. 6e).

Two-photon microscopy has become an indispensable tool for imaging due to its deep imaging depth and high resolution.^97–99^ An ultracompact two-photon microscopy is hard to build because an objective lens that exhibits high NA is also bulky. In addition, a fluorescence imaging system should satisfy the working range in at least two wavelengths, one for the excitation and one for emission of the fluorescence molecules, which are relatively spectrally far apart. A compact two-photon microscope system (Fig. 6f) has been demonstrated by using a thin double-wavelength metasurface objective lens.^100^ A birefringent dichroic dielectric metasurface was designed, and the wavelengths of excitation and emission have the same focal distance from the metasurface that works as the objective lens. The metalens has a diameter of 1.6 mm, focal length of 1.386 mm and NA of 0.5, which is the same as that of the conventional objective lens. The image quality achieved by the designed metalens system was comparable to that of a conventional objective lens (Fig. 6g).

Metalenses have been integrated into an endoscopic optical imaging system.^101^ Endoscopic OCT has deep (millimeter-scale) tissue-imaging capability, so the technique has been regarded as a promising tool for monitoring tissues or diseases. Existing OCT catheters use a lens-prism that has a graded refractive index, or a ball lens.^102^ These catheters must have small volume, and are therefore difficult to manufacture, and suffer from chromatic aberration and poor focus. Metasurface-based fiber optic catheters with metalenses achieved high-resolution imaging and extended depth of focus (DOF) when connected to a Fourier-domain OCT system. Effective DOFs of 211 μm and 315 μm were achieved; these are larger than that of a conventional achromatic lens (90 μm) that has the same NA. This DOF is only feasible using a system that uses a metalens and has great potential in miniaturization while retaining high optical quality. *In vivo* and *ex vivo* endoscopic OCT images obtained using the metalens endoscope generate a very clear and high quality optical image (Fig. 6h).

### 3.2 Super-resolution imaging

Super-resolution imaging techniques, also called optical nanoscopy, have attracted great interest recently. The key is to break the diffraction limit, which is a physical resolution barrier defined as *λ*/2NA.^103^ The developers of stimulated emission depletion microscopy (STED)^104^ and single-molecule localization microscopy (SMLM)^105–108^ won the Nobel Prize by exploiting a mechanism to control the switching of fluorophores. At a similar time, structured illumination microscopy (SIM) was developed.^109^ Today SIM is considered as a powerful and versatile super-resolution technique due to its resolution improvement, fast acquisition speed and system flexibility. In general, conventional wide-field fluorescence microscopes can be integrated to SIM microscope, hence commercially available fluorescence probes can be used for SIM microscopes. This field has undergone extraordinary development recently, and many SIM techniques have been reported.^109,110^

SIM overcomes the diffraction limit by using spatial frequency mixing to encode structural illumination patterns that have high spatial frequencies into a sample. High-resolution information can be recovered from the low-frequency information, which is dissipated by conventional imaging systems. However, SIM techniques are theoretically restricted in their spatial resolution,^111^ since the resolution in SIM is defined by the spatial frequency of illumination patterns that are also limited by diffraction. A proposed SIM with nonlinear modalities may overcome the theoretical resolution limit by using illumination with sinusoidal light patterns that have high intensity.^112,113^ The emission result has a harmonic pattern, which deviates from sinusoidal because fluorophores in the sample become saturated. Even though the higher harmonics extend the optical transfer function, the high intensity of illumination to achieve saturation can cause photobleaching and phototoxicity.

**Fig. 7:**
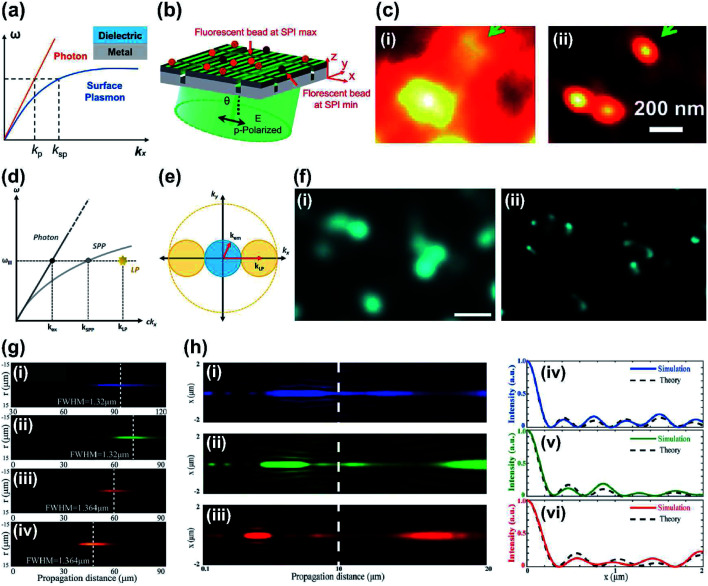
(a) Schematic of dispersion plot. Surface plasmon (SP) at the interface of metal and dielectric layer represents large wavevector compared to the photon at the same frequency, so the SP has potential to achieve sub-diffraction illumination patterns. (b) Schematic of PSIM with designed metasurface with slits. (c) Imaging results of PSIM using fluorescence beads (diameter: 100 nm). Compared to (i) the conventional image, (ii) PSIM improved resolution by up to 2.6 times. (d) Illustration of the dispersion plot of SP and localized plasmon (LP). (e) Schematic of spatial frequency showing that the LP may improve the spatial resolution of the image high-*k* information can be captured, then shifted into the observable regime of optical transfer function. (f) Experimental demonstration of LPSIM using fluorescent beads (diameter: 50 nm). (i) The blurred image in conventional wide-field microscope was clearly resolved (ii) with LPSIM (scale bar: 500 nm). (g) Experimental demonstration of light distributions of SOLs along *z* axis at wavelengths (i) 405 nm, (ii) 532 nm, (iii) 632.8 nm and (iv) 785 nm. (h) Calculated results of achromatic SOLs along *z* axis at wavelengths of (i) 473 nm, (ii) 532 nm and (iii) 632.8 nm, and their (iv–vi) respective intensity profiles at *z* = 10 μm. (a–c) Reprinted with permission from ref. 115. Copyright 2014 American Chemical Society. (d–f) Reprinted with permission from ref. 117. Copyright 2017 American Chemical Society. (g) Reprinted with permission from ref. 138. Copyright 2015 John Wiley & Sons, Inc. (h) Reprinted with permission from ref. 143. Copyright 2019 The Optical Society.

A different way of making high frequency illumination patterns, like the nonlinear saturated SIM, is sought. Various designs that use SPPs,^114^ which can be spatially patterned at very high spatial frequency, have been demonstrated using metallic-based metasurfaces. The spatial frequency *k* is dependent on the angular frequency *ω*, because of their relationships with the speed of light *c*. However SPP can have bigger values of *k* than the free-space light (Fig. 7a). Furthermore, interference between two counter propagating SPPs can generate fine stripe patterns that have great potential to be implemented in SIM to further increase its resolution. The background fluorescence also can be suppressed because SPP intensity decays exponentially along the axis away from the surface.

Plasmonic structured illumination microscopy (PSIM) has emerged as a powerful super-resolution method.^115^ The PSIM (Fig. 7b) uses a metasurface that includes a periodic slit arrangement. The metasurface was fabricated to have slit array patterns on a glass substrate, and silver (Ag) was used to generate surface plasmons at an Ag/dielectric interface in visible wavelength with low loss. The illumination light was coupled into the metasurface and SPPs generated standing waves at the metal boundaries with specific angle of incidence for efficient SPP coupling. Plasmon waves were launched on both sides of the slits and diffraction-unlimited interference patterns were formed. The phase of the interference patterns could be controlled by making small adjustments to the illumination pattern's angle of incidence. Use of high-frequency patterns on the metasurface yielded resolution improvement by a factor of 2.6 (Fig. 7c).

Localized surface plasmon assisted structured illumination microscopy (LPSIM) is a one-step advanced imaging technique. It has been proposed theoretically by using localized plasmons (LPs) generated on a metasurface composed of an array of nanostructure.^116^ Localized surface plasmon resonances can be defined as coherent oscillations of electrons excited by electromagnetic radiation in metallic nanostructures. LPs are basically bound to the metal–dielectric interface, so the size of structures is determined by the shape of the interface. LPSIM exploits plasmonic excitations from an engineered pattern from a 2D array on a metasurface; the array was used as a structured illumination pattern. Patterns from LPs can obtain unlimited spatial frequency fundamentally and can thus acquire more images that have much higher resolution compared to SIM and PSIM. *k* can also be increased, so further improvement in resolution is possible (Fig. 7d and e).

The first numerical demonstration of LPSIM increased resolution by a factor of three compared to conventional fluorescence microscopy. The metasurface that was implemented consisted of a hexagonal array of Ag nanodiscs on a glass dielectric. The nanodiscs had diameter of 60 nm, thickness of 60 nm and were set at a pitch of 150 nm. The phase of patterns was shifted easily by changing the angle of incidence. LPSIM was experimentally demonstrated using finely-structured near-field excitation patterns that were obtained from localized surface plasmons that used plasmonic metasurfaces.^117^ Changeable illumination patterns were formed using an LPSIM substrate with an array of Ag nanodiscs. The illumination patterns could be controlled by changing the angle of incidence and polarization of the incidence laser light. A custom polarizer plate and a 2D scanning mirror system were integrated for LPSIM measurement setup, and nine images can be collected and used to reconstruct a super-resolution image. LPSIM achieved wide-field super-resolution imaging down to 75 nm, which is a finer resolution than conventional SIM (Fig. 7f).

LPSIM can be combined with a near-field microlens to increase resolution even if the measurement setup uses an objective lens that has low NA.^118^ A metasurface that uses dielectric microspheres that have high refractive index showed strongly increased imaging resolution due to near-field coupling, and demonstrated that it improved the effective NA of the imaging system.^119–121^

In a similar setup, LPSIM was combined with dielectric microspheres that had been optically trapped using an IR beam to achieve precise position control. A plasmonic metasurface that included 60 nm Ag discs in a hexagonal array was used for illumination of patterns, and the acquired image set was used for reconstruction. Compared to conventional microscopy, this LPSIM with integrated microspheres achieved seven times finer resolution. Similarly, a hyperbolic metamaterial was combined with the structured illumination technique.^122^ Sub-diffraction-limited illumination patterns can be projected due to its highly dispersive optical characteristics.^123^ An Ag/SiO_2_ hyperbolic metamaterial was used in measurement and one-dimensional resolution improvement down to 84 nm was achieved.

LPSIM has been further developed and has achieved highly improved spatiotemporal resolution for observation of cell dynamics.^124^ A high-speed camera and multiple lasers were synchronized for 40 Hz video speed imaging. A newly-designed sapphire metasurface was used as an illumination platform; images with a wide field of view of 28 × 28 μm^2^ were acquired. Experimental imaging reached resolution of <50 nm, which corresponds to *λ*/11.

To obtain super resolution images implementing SIM microscopy, various structured patterns have been used in addition to periodic patterns from plasmonic metasurfaces: a single focal spot,^125^ multifocal spot^126–128^ and random speckles.^129–133^ Various structured patterns have been formed by using bulky and expensive equipment systems such as SLM and digital micro-mirror devices. Recently, the resolution of fluorescence microscopy has been refined by using encoded patterns from all-dielectric metasurfaces.^134^ This approach shows promise as a substitute illumination optical device with high efficiency and small size. The encoded patterns from dielectric metasurfaces can be formed specifically according to the design. The metasurfaces used were designed based on the PB phase. The unit-cell orientation manipulates the phase distribution, and the desirable illumination patterns can be encoded. A diffuser pattern was used for the experiment; the achieved resolution was <155 nm after reconstruction,^135^ which is 1.71 times finer than achieved by conventional fluorescence microscopy.

Super-oscillatory lens (SOL) is another promising technique for far-field and label-free super-resolution imaging.^136^ Superoscillation describes the phenomenon in which bandlimited function can have local oscillations that can be faster than the oscillations of the fastest Fourier components. Superoscillation allows light to be focused on a small spot; this ability can be useful for subwavelength super-resolution focusing and imaging. The structures of SOLs can be designed using phase-type metasurface masks,^137,138^ SLM,^139^ and binary amplitude masks,^140,141^ to precisely control the amplitude or phase of light. SOLs that use metasurfaces have demonstrated the capability of SOLs as imaging tools by confining the point spread function below the subwavelength scale. SOLs also have the advantage of being non-invasive. However, most SOLs have limitations of narrowband working regime and a dependence on wavelength.^140,142^ To solve these problems, ultra-broadband SOL that benefits from the excellent ability of metasurfaces to control electromagnetic waves was suggested; the method was capable of far-field sub-diffraction focusing (Fig. 7g) and had good imaging results.^138^

Chromatic aberration remains a problem in imaging using lenses that use metasurfaces. Recently, broadband achromatic SOLs have been fabricated using dielectric materials.^143,144^ Dielectric metasurfaces with high refractive index precisely controlled the phase of the light and also achieved a single-layer structure, which can yield compact SOLs. These SOLs largely eliminated chromatic aberration in experiments with input sources that had different wavelengths (Fig. 7h). The light through SOLs can focus below the diffraction limit, and a low-cost, compact optical system can be constructed.

### 3.3 Computational imaging

Computational imaging (CI), emerged with fast computing platforms such as multi-core CPUs. CI techniques indirectly form images from measurements by applying a recovery algorithm. Fundamentally, CI depends on captured images and post-processing techniques from a series of inputs under different illumination or optical conditions. Thus, CI can be regarded as a combination of computation and optical encoding. Super-resolution imaging techniques presented in the previous section are also included in CI. This section will introduce other applications of CI that use metasurfaces: ghost imaging, complex optical field imaging and full-color imaging.

Ghost imaging (GI) is an indirect imaging approach that is used to acquire information about an object. This is done by correlating two light beams with fluctuating intensity.^145,146^ Computational GI simplifies the experimental setup by requiring only a single-pixel detector.^147,148^ The correlation between the object beam and reference beam is replaced by the temporal correlation in the intensity captured by the single-pixel detector and the computational patterns for the recovery of the object image.

**Fig. 8:**
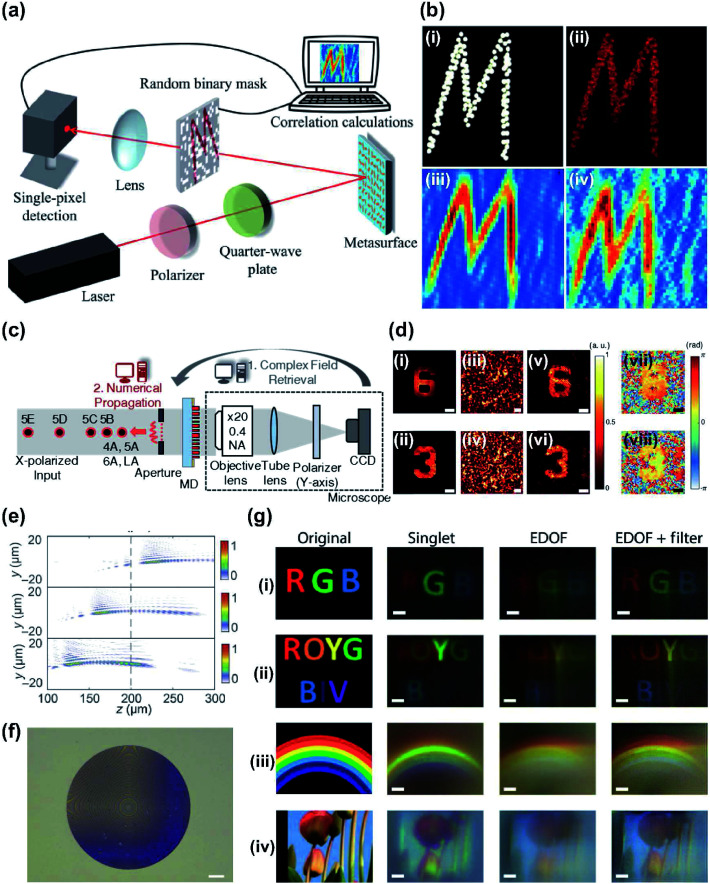
(a) Schematic of computational GI setup using designed metasurface. (b) Calculated and measured results using GI algorithm (compressive GI). (i) Original and (ii) holographic image as reconstructed from different numbers of measurements: (iii) 2601 and (iv) 668. (c) Experimental setup of computational complex optical field imaging with diffuser that uses metasurface, and computational steps at various distances. (d) Experimentally reconstructed results of the 1951 USAF resolution target. (i and ii) amplitude Captured using conventional microscope and (iii and iv) scattered light patterns from diffuser that uses a metasurface. Retrieved (v and vi) amplitude images and (vii and viii) phase images. (e) Calculated intensity profiles of the extended depth of field metasurface along the *z* axis at wavelengths 400 nm, 550 nm and 700 nm. (f) Optical image of fabricated extended depth of field metasurface (scale bar: 25 μm). (g) Retrieved full-color images captured under white illumination with different-colored objects, (i) RGB, (ii) ROYGBIV, (iii) rainbow and (iv) sky and colorful flowers. (a and b) Reprinted with permission from ref. 149. Copyright 2017 The American Association for the Advancement of Science. (c and d) Reprinted with permission from ref. 159. Copyright 2018 The Optical Society. (e–g) Reprinted with permission from ref. 163. Copyright 2018 The American Association for the Advancement of Science.

Single-pixel computational GI has been achieved by integrating computational GI with metasurfaces.^149^ A highly efficient, broadband reflective metasurface with an Au–MgF_2_–Au Fabry–Perot cavity was designed and fabricated. The metasurface reflected a holographic image that was projected onto a random binary mask, and a single-pixel detector was implemented to detect the transmitted light (Fig. 8a). Hundreds of measurements could be performed in this way, due to the holographic image recovery by correlation calculation; the clarity of the image increases the higher the number of measurements performed.^147^ In addition, the helicity-dependent functionality of metasurfaces allows recovery of spatially-inverted holographic images according to the polarization state of incident light (Fig. 8b). The designed metasurface showed helicity-dependent ghost images and can be used in security applications such as optical encryption that uses GI.

Metasurfaces have been used in complex field imaging to extract hidden information from complicated speckle patterns. Because a complex speckle pattern is generated when coherent light passes through a scatterer, it is difficult task for imaging through scattering media. Review articles^150^ and methods such as OCT,^151^ speckle correlation using the memory effect,^152,153^ wave front engineering,^154^ the transmission matrix^155^ and speckle-correlation scattering matrix^156^ have been introduced to overcome the difficulties in imaging through scattering media.

Speckle-based computational imaging has the advantage that it can acquire hidden information by using a conventional optical system. Various speckle-based techniques have been proposed to measure a complex field through a scattering medium. However, they have unstable optical properties, fluctuation and short memory-effect range, which can be major drawbacks.^65,157,158^ Metasurfaces were used as scattering media when designed to act as an optical diffuser for complex field and 3D imaging.^159^ The metasurface-based diffuser (MD) consisted of amorphous silicon meta-atoms, and designed wavelength was 850 nm with independent 2π phase coverage for both *x*- and *y*-polarized light. The complex fields can be retrieved by using the computed *T* matrix of the metasurface and the intensity of a measured speckle pattern. The designed MD operated as a phase mask to achieve a uniform light scattering, which needs a uniform amplitude in the Fourier domain, resulting in several advantages such as reduction of the time-consuming characterization process in a single calculation. This technique that uses metasurfaces does not require changes in the measurement setup or conditions, because the new *T* matrix can be easily calculated. Complex fields with objects were retrieved experimentally (Fig. 8c) by measuring amplitude samples and by holographic imaging (Fig. 8d). Reliable reproducibility, stability and high noise tolerance were demonstrated. Computational methods that exploit speckling by metasurfaces are compact and compatible with conventional system, and have been extended to various applications in diagnostics,^160^ holography^161^ and encryption.^162^

Metasurface optics and computational imaging have been combined with CI to achieve full-color imaging.^163^ Metalenses for imaging over the full visible spectrum are being actively studied. One of the main challenges is the implementation of achromatic metasurface lenses under broadband white light illumination (Section 2).^76^ Optical metalenses with computational post processing have demonstrated full-color achromaticity under white light source by using a single metasurface and a digital filter. Chromatic aberrations shifted the image plane as the wavelength was changed. The resulting image captured in different image planes was blurred; this defect was mitigated by the extended depth of focus optics in the visible regime^164,165^ (Fig. 8e and f). The metasurface engineered the point spread function to be extended so that it did not shift as wavelength changed. Metasurfaces provided design freedom for combining wavefront coding and lens into a compact element without the need to fabricate a secondary phase mask. A linear Wiener filter was applied to a computational imaging system to retrieve the desired full-color images.^163^ To retrieve the desired image, the advantages of wavelength invariance and lack of zeros in the broad spatial frequency simplified computational reconstruction. Imaging was demonstrated under both discrete illumination and broadband illumination. Measurement and post processing greatly reduced the chromatic aberration compared to the normal imaging result captured by singlet (Fig. 8g). The same results can be acquired under broadband illumination condition. In-focus full-visible spectrum imaging was achieved.

### 3.4 Functional imaging: polarization and hyperspectral imaging

**Fig. 9:**
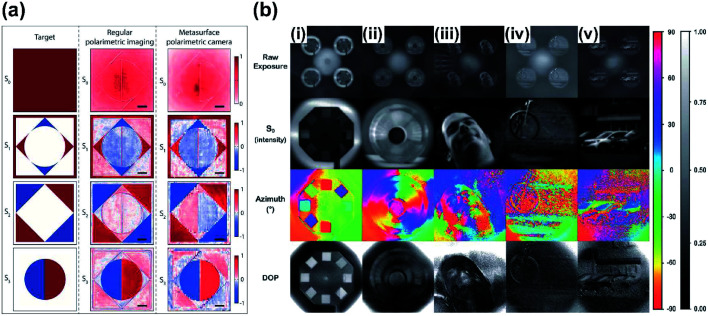
Polarimetric imaging results using metasurface polarization camera. The target image (polarization mask) was captured using conventional polarimetry and metasurface polarimetric camera (scale bar: 100 μm). (b) Full-stokes polarization images based on metasurface polarization camera without any conventional polarization optics and moving components. (i–iii) Indoor and (iv and v) outdoor photograph with raw exposure, S_0_, azimuth of polarization ellipse, and degree of polarization (DOP). (a) Reprinted with permission from ref. 169. Copyright 2018 American Chemical Society. (b) Reprinted with permission from ref. 170. Copyright 2019 The American Association for the Advancement of Science.

Polarization analysis and functional imaging application such as polarimetric imaging are made possible by the ability of metasurfaces to manipulate polarization and phase. Measurement of the polarization of light can be exploited to achieve polarimetric imaging, which can obtain information such as shape and texture of a surface, and orientation of the light.^166^ Mapping of polarization states has been reported.^167,168^ Polarimetric imaging requires measurement of the intensity at different polarizations, then represents the results as a full Stokes vector for estimation. However, the use of polarization filters or apertures has restricted the theoretical efficiency limit to 50%. The ability of metasurfaces to control phase and polarization with high efficiency suggests that they can be used in polarimetric imaging. A dielectric metasurface mask for polarization cameras (PCs) can measure the Stokes parameters.^169^ A single dielectric layer did not require polarization filters, and its use of polarization splitting and focusing allowed it to exceed the theoretical efficiency. Metasurfaces fully measure the polarization state by acquiring intensities under different conditions by projection of linear and circularly polarized light without the need to change optical components such as half- and quarter-wave plates. Stokes parameters can be easily acquired using a simple optical setup that does not require polarization filtering. The polarimetric imaging results (Fig. 9a) agreed well with the results of conventional polarimetric imaging results and had advantages of efficiency and simplicity in the system.

A single-shot polarization camera has been reported.^170^ Matrix Fourier optics was introduced and applied to characterize polarization in diffractive optics. Dielectric metasurfaces were designed to realize diffraction gratings, which each act as polarizers that have arbitrarily-chosen polarization states. This arbitrary polarization set can be achieved by single unit cell, so the optical device can be compact. Existing optical systems integrate designed metasurfaces with a full-Stokes polarization camera and can acquire polarization information (Fig. 9b) without polarization optics.

Hyperspectral imaging extracts information about molecular composition. This can be used in sensing applications if combined with imaging optics.^171,172^ A dielectric metasurface that supported bound states in the continuum (BIC)^173^ with extremely high quality factor was fabricated, then used in ultrasensitive hyperspectral imaging for biodetection.^174^ The dielectric metasurface that was used had nano-resonators with low absorption losses^52,175^ which supported BIC modes that have a sharp resonance peak and a strong confined light field. Therefore, high sensitivity to changes in refractive index in response to varying identities and positions of nearby molecules can be achieved in visible and NIR regimes.^176^ The designed dielectric metasurface showed tunable BIC resonances in the NIR region, so hyperspectral images to sense biomolecules can be acquired in a single measurement without the need for a scanning or spectrometer system. A supercontinuum laser source was combined with a bandpass filter; the imaging optics used a CMOS camera to record images at each illumination wavelength. The full spectral response over areas from dielectric metasurface was captured by a sensor array and can be translated to form a map of resonances from each sensor.^177^ The maps were used for comparison with reference maps, and spectral shift information was finally acquired. The demonstration of compatibility of dielectric metasurfaces with biosensing showed improved analytical characterization and evaluation of the information captured by each individual pixel of CCD and resonance shift map. The result showed that high resolution spectral analysis and hyperspectral imaging were possible with elimination of bulky and expensive instrumentation, and that use of metasurfaces can increase the sensitivity.

## 4. Conclusions and outlook

Metasurfaces have revolutionized many optical fields by replacing refractive optics and by revealing new features that traditional optical systems had not detected. The metasurfaces' ability to control the wavefront of light enables realization of planar optics, and practical applications and technology in optical regimes have been developed. Especially, metalenses are being rapidly developed for use in imaging. Various technologies have been developed, from replacement of conventional optical lenses to development of functional optical components such as polarizers. Actual production and system applications can be expected soon. In addition, metasurfaces can include various functions, so these surfaces have applications to super-resolution imaging, polarimetric imaging, holographic imaging and other sensing fields.

In this review, we have focused on use of metasurfaces in the imaging and have summarized several methods. First, planar optical components, *i.e.*, metalenses, were discussed. Metalenses have shown great potential as a replacement for conventional optical components and as components of miniaturized optical systems. Research on metalenses has made breakthroughs, such as attaining wide FOV of up to ±90° and eliminating chromatic correction in the visible and NIR regimes. Tunable metalenses present another exotic functionality, which is an ability to tune their output optical wavefront.

Second, functional imaging applications that use metasurfaces were discussed. Uses of metasurfaces are not simply confined to lens design and verification but have begun to be applied and used in optical equipment in biology and materials science. Commercialized imaging equipment such as confocal microscopy, OCT and two-photon microscopy are prime examples of how lenses that use metasurfaces can be applied to existing systems, which can be further extended. Metasurfaces have also been adopted in successful imaging platforms for super-resolution imaging, computational holography, and multicolor imaging system, as well as other functional imaging techniques such as polarimetric imaging and hyperspectral imaging. This wide range of applications shows the potential of functional optical devices and platforms. In addition, metasurfaces are expected to expand their influence by combining with artificial intelligence,^178^ 3D holographic display,^179^ phase imaging^180^ and detecting technology.^181^

Metasurfaces have been demonstrated as a new paradigm that can displace optical components. Methods to fabricate metasurfaces are inexpensive; large-scale fabrication methods like nanoimprint lithography and deep UV lithography can be considered due to their advantages in mass production, low cost and high speed. Use of nanoimprint lithography to produce dielectric metasurfaces has high productivity at competitive cost,^182,183^ and demonstrates the potential for quick adoption.

Metasurfaces are also expected to open new avenues in the fields of meta-optics.^184^ Metasurfaces can replace refractive and diffractive optics in widely-used items such as smartphones and laptops, and in special imaging devices like virtual reality and wearable optics.^185^ Multiple optical functions can be encoded in the metasurface; this ability will lead to improvement of optical components and instruments,^186^ and in the future may allow development of single devices that can replace bulky and expensive elements like birefringence components or electronic devices for structured illumination.

## Conflicts of interest

There are no conflicts to declare.

## Supplementary Material
